# Efficacy and safety of carbon ion radiotherapy for bone sarcomas: a systematic review and meta-analysis

**DOI:** 10.1186/s13014-022-02089-0

**Published:** 2022-10-25

**Authors:** Meng Dong, Ruifeng Liu, Qiuning Zhang, Hongtao Luo, Dandan Wang, Yuhang Wang, Junru Chen, Yuhong Ou, Xiaohu Wang

**Affiliations:** 1 Institute of Modern Physics, Chinese Academy of Sciences, Lanzhou, People’s Republic of China; 2 The First School of Clinical Medicine, Lanzhou University, No.1, Donggang West Road, Lanzhou, 730000 People’s Republic of China; 3 Department of Postgraduate, University of Chinese Academy of Sciences, Beijing, People’s Republic of China; 4 Heavy Ion Therapy Center, Lanzhou Heavy Ions Hospital, Lanzhou, People’s Republic of China

**Keywords:** Particle, Carbon ion radiotherapy, Bone sarcoma, Systematic review, Meta-analysis

## Abstract

**Objective:**

This study aimed to systematically evaluate and conduct a meta-analysis of the efficacy and safety of carbon ion radiotherapy for bone sarcomas.

**Methods:**

We searched for articles using the PubMed, Embase, Cochrane Library, and the Web of Science databases from their inception to January 12, 2022. Two researchers independently screened the literature and extracted data based on the inclusion and exclusion criteria. Statistical analyses were performed using STATA version 14.0.

**Results:**

We searched for 4378 candidate articles, of which 12 studies were included in our study according to the inclusion and exclusion criteria. Of the 897 BSs patients who received carbon ion radiotherapy in the studies, 526 patients had chordoma, 255 patients had chondrosarcoma, 112 patients had osteosarcoma, and 4 patients had other sarcomas. The local control rate at 1, 2, 3, 4, 5, and 10 years in these studies were 98.5% (95% confidence interval [CI] = 0.961–1.009, *I*^2^ = 0%), 85.8% (95% CI = 0.687–1.030, *I*^2^ = 91%), 86% (95% CI = 0.763–0.957, *I*^2^ = 85.3%), 91.1% (95% CI = 0.849–0.974), 74.3% (95% CI = 0.666–0.820, *I*^2^ = 85.2%), and 64.7% (95% CI = 0.451–0.843, *I*^2^ = 95.3%), respectively. The overall survival rate at 1, 2, 3, 4, 5, and 10 years in these studies were 99.9% (95% CI = 0.995–1.004, *I*^2^ = 0%), 89.6% (95% CI = 0.811–0.980, *I*^2^ = 96.6%), 85% (95% CI = 0.750–0.950, *I*^2^ = 89.4%), 92.4% (95% CI = 0.866–0.982), 72.7% (95% CI = 0.609–0.844, *I*^2^ = 95.3%), and 72.1% (95% CI = 0.661–0.781, *I*^2^ = 46.5%), respectively. Across all studies, the incidence of acute and late toxicities was mainly grade 1 to grade 2, and grade 1 to grade 3, respectively.

**Conclusion:**

As an advanced radiotherapy, carbon ion radiotherapy is promising for patients with bone sarcomas that are unresectable or residual after incomplete surgery. The data indicated that carbon ion radiotherapy was safe and effective for bone sarcomas, showing promising results for local control, overall survival, and lower acute and late toxicity.

***PROSPERO registration number*:**

CRD42021258480.

**Supplementary Information:**

The online version contains supplementary material available at 10.1186/s13014-022-02089-0.

## Introduction

Bone sarcomas (BSs) are malignant tumors originating in human mesenchymal tissue, have a low incidence, complex pathological types, and significant heterogeneity, and are difficult to treat. BSs include osteosarcomas, chondrosarcomas, and chordomas. The traditional treatment for sarcomas is aggressive surgery; upon R0 resection, local recurrence (LR) incidence at 5 years has been reported in 6% of patients [1–3]. However, the complete resection of some sarcomas might not be possible in patients with anatomical complexity; the R2 resection LR rate at 5 years has been reported as 38% [1–4]. In this case, radiotherapy (RT) is an important potential treatment strategy for some patients who have sarcomas that are unresectable or residual after incomplete surgery [5, 6].

Most types of BSs are known to be resistant to radiation, requiring higher dose irradiation to gain adequate local control (LC) [3]. However, traditional radiation therapy, such as photon therapy, has a limited ability to achieve lethal dose irradiation due to the proximity of some sarcomas to organs at risk. In the recent years, advanced radiation modalities including protons and carbon ions have been developed. They can deposit a dose in the “Bragg peak” region providing a more favorable dose-distribution compared to photons, which can deliver a higher dose to the tumor area, while protecting the surrounding tissues and organs. In addition, they have a significant relative biological effectiveness (RBE), especially carbon ions, which exert a greater killing effect on tumor cells than protons. As one of the more advanced radiotherapy modalities, carbon ion radiotherapy (C-ion RT) is a promising treatment strategy for sarcomas [7].

In Japan, C-ion RT was carried out in 1994 for treating all kinds of cancer, including various unresectable BSs [7]. For a long time, the clinical studies of carbon ion therapy for BSs have been mainly reported in case series; however, the sample size was small, and the efficacy and safety were not clear and definite. Therefore, the aim of this study was to systematically evaluate and analyze the comprehensive evidence for C-ion RT treatment of BSs and to provide the latest evidence for C-ion RT clinical treatment, guideline formulation, and policy implementation.

## Materials and methods

### Literature identification

This systematic review and meta-analysis followed the Preferred Reporting Items for Systematic Reviews and Meta-analysis (PRISMA) guidelines, and the review protocol was registered in PROSPERO (CRD42021258480).

### Search strategy

Our search strategy was determined according to the PRISMA guidelines and recommendations [8]. We searched for articles using the Cochrane Library, Embase, PubMed and Web of Science databases, from their dates-of-inception to 12 January 2022. Notably, only the literature written in the English language was considered. The search terms were as follows: (“Sarcoma OR Sarcoma* OR Soft Tissue Sarcoma* OR Epithelioid Sarcoma* OR Spindle Cell Sarcoma*” AND “Heavy Ion Radiotherapy OR Heavy Ion Radiotherapies OR Heavy Ion Therapy* OR Heavy Ion Radiation Therapy OR Carbon Ion Radiotherapy OR Carbon Ion Therapy* OR Carbon Ion Radiation Therapy OR C-ion therapy OR hadron OR particle OR charged particle”). Simultaneously, the references included in the study were traced back to obtain relevant information not found in the above retrieval.

### Inclusion and exclusion criteria

All the retrieved articles were independently screened by two researchers (MD, QZ). The studies were included as per the following criteria: (a) studies wherein the patients were clinically or pathologically diagnosed with primary or recurrent BSs and (b) clinical studies reporting survival outcomes and toxicity incidence in patients who were treated with C-ion RT. The survival outcome data of these studies were required to include both the overall survival (OS) rates and LC from the initial diagnosis. The exclusion criteria were (a) studies reporting patients from treatment only with photons, protons, brachytherapy, and other particles; (b) duplicate publications; (c) case reports, reviews, meta-analyses, abstracts, letters, comments, and protocols; (d) re-irradiation studies; (e) lack of detailed data; (f) clinical studies with fewer than 10 patients; and (g) other irrelevant topics.

### Data extraction

Literature screening and data extraction were performed by two reviewers (YW and QZ) independently from the selected studies, and the results were checked by a third reviewer (DW). If there was any disagreement, the three investigators discussed together until a consensus was reached. Data extraction included the following: (a) first author, journal, publication year, country, research institution, study design, and study period; (b) number of patients, age, sex, tumor site, histology, tumor status, tumor volume, total treatment dose, fractions, fraction dose, and follow-up time; (c) the primary outcome was OS, and secondary outcomes were LC, toxicity, and LR; and (d) evaluation indicators of quality and bias assessments.

### Quality and bias assessments

In our systematic review, each included article was a case series, which were evaluated using the Joanna Briggs Institute critical appraisal tool for case series [9]. The literature quality and bias assessments were independently completed by two researchers (QZ and MD), and disputes were resolved by a third reviewer (DW) with answers as yes, no, unclear, or not applicable. The evaluation indicators and outcomes are shown in Table 1.

**Table 1:** Assessment of risk of bias in included studies

Study	Criterion									
	A	b	c	d	e	f	g	h	i	j
*Japan*										
Shiba 2021 [10]	Yes	Yes	Yes	Yes	No	Yes	Yes	Yes	No	Yes
Mohamad 2018 [11]	Unclear	Yes	Yes	Yes	No	Yes	Yes	Yes	No	Yes
Imai 2017 [12]	Yes	Yes	Yes	Yes	No	Yes	Yes	Yes	No	Yes
Imai 2016 [13]	Yes	Yes	Yes	Yes	No	Yes	Yes	Yes	No	Yes
Matsunobu 2012 [14]	Yes	Yes	Yes	Yes	No	Yes	Yes	Yes	No	Yes
Imai 2011 [15]	Yes	Yes	Yes	Yes	No	Yes	Yes	Yes	No	Yes
Mizoe 2009 [16]	Yes	Yes	Yes	Yes	No	Yes	Yes	Yes	No	Yes
*Germany*										
Mattke 2018 [17]	Yes	Yes	Yes	Yes	No	Yes	Yes	Yes	No	Yes
Uhl 2014 [18]	Unclear	Yes	Yes	Yes	No	Yes	Yes	Yes	No	Yes
Uhl 2014 [19]	Unclear	Yes	Yes	Yes	No	Yes	Yes	Yes	No	Yes
Combs 2009 [20]	Unclear	Yes	Yes	Yes	No	Yes	Yes	Yes	No	Yes
*China*										
Wu 2019 [21]	Yes	Yes	Yes	Yes	No	Yes	Yes	Yes	No	Yes

(a) Were there clear criteria for inclusion in the case series?; (b) Was the condition measured in a standard, reliable way for all participants included in the case series?; (c) Were valid methods used for identification of the condition for all participants included in the case series?; (d) Did the case series have consecutive inclusion of participants?; (e) Did the case series have complete inclusion of participants?; (f) Was there clear reporting of the demographics of the participants in the study?; (g) Was there clear reporting of clinical information of the participants?; (h) Were the outcomes or follow-up results of cases clearly reported?; (i) Was there clear reporting of the presenting sites’/clinics’ demographic information?; (j) Was statistical analysis appropriate?

### Statistical analysis

Descriptive statistics were used to summarize the baseline variables and the incidence of toxicity. The data descriptions included frequencies and percentages for dichotomous data, and means with standard deviations or medians with interquartile ranges for continuous data. The case series studies were conducted under different conditions. Thus, we used a random effects (RE) model to provide an overall summary estimate. We computed the proportion with 95% confidence intervals (CIs) to estimate the effect sizes for continuous outcomes. All the analyses were performed using STATA version 14.0 (StataCorp, College Station, Texas).

## Results

### Study selected and characteristics

**Fig. 1:**
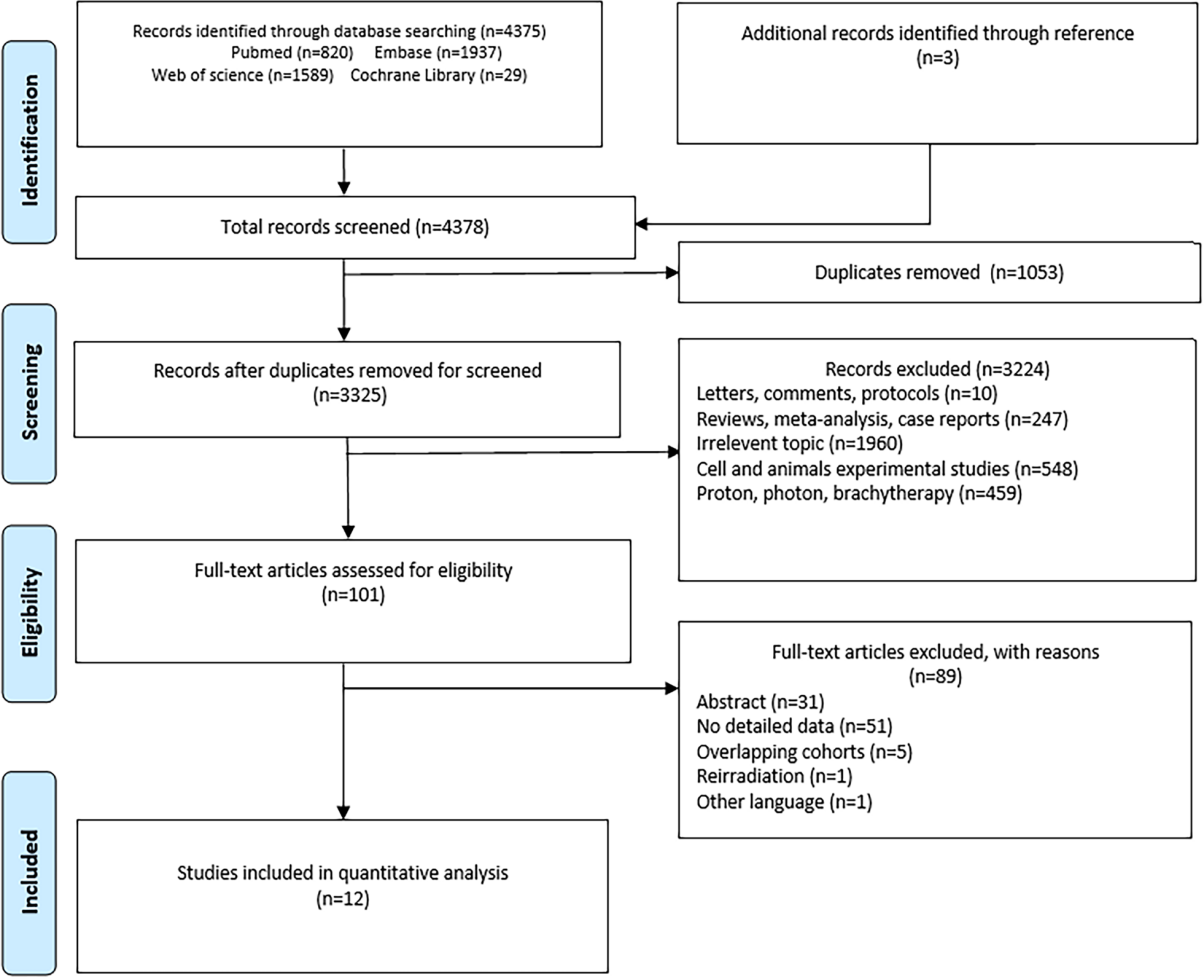
Search results per the PRISMA guidelines

As shown in Fig. 1, the systematic searches produced 4378 potential articles for inclusion. After title and abstract review, we removed 1053 duplicates, resulting in 3325 remaining reports. In total, 101 related studies were screened for full-text article eligibility. We eliminated another 89 items, including 31 abstracts, 51 with no detailed data, 5 overlapping cohorts, 1 re-irradiation, and 1 other language (German), and eventually included 12 articles. These 12 studies originated from three countries, Japan n = 7), Germany (n = 4), and China (n = 1) [10–21]. The study design included nine retrospective studies, two prospective studies, and one phase I/II or II trial (Table 2). Of the 897 BSs patients who received C-ion RT in the studies, 526 patients had chordoma, 255 patients had chondrosarcoma, 112 patients had osteosarcoma, and 4 patients had other conditions. These studies reported the survival and toxicity. Overall, the median sample size was 75.5 patients (range, 17–188), median age ranged from 16 to 67 years, and median follow-up time ranged from 21.8 to 91 months (Table 2).

**Table 2:** Baseline characteristics of included studies

Study	Institution	Study type	Outcomes	Period	No. of patients	Median age (year)	Male/female	Median follow-up (month)
Shiba 2021 [10]	Japan (GHMC)	Prospective	Survival, toxicity	2011–2019	53	67 (14–84)	32/21	36.9 (4.4–96.4)
Mohamad 2018 [11]	Japan (NIRS)	Retrospective	Survival, toxicity	1996–2014	26	16 (11–20)	18/8	32.7 (1.2–248)
Imai 2017 [12]	Japan (NIRS)	retrospective	Survival, toxicity	2000–2012	73	57 (17–77)	31/42	49.4 (6.4–146.4)
Imai 2016 [13]	Japan (NIRS)	retrospective	Survival, toxicity	1996–2013	188	66 (26–87)	128/60	62 (6.8–147.5)
Matsunobu 2012 [14]	Japan (NIRS)	prospective	Survival, toxicity	1996–2009	78	41 (11–83)	49/29	24 (2–166)
Imai 2011 [15]	Japan (NIRS)	retrospective	Survival, toxicity	1996–2007	95	66 (30–85)	68/27	42 (13–112)
Mizoe 2009 [16]	Japan (NIRS)	phase I/II or II	Survival, toxicity	1995–2007	33	47 (16–76)	14/19	53 (8–129)
Mattke 2018 [17]	Germany (HIT)	retrospective	Survival, toxicity	2009–2014	79	46	32/47	43.7
Uhl 2014 [18]	Germany (HIT)	retrospective	Survival, toxicity	1998–2008	155	48 (15–85)	76/79	72 (12–165)
Uhl 2014 [19]	Germany (HIT)	retrospective	Survival, toxicity	1998–2008	79	45 (16–81)	39/40	91 (3–175)
Combs 2009 [20]	Germany (HIT)	retrospective	Survival, toxicity	1997–2007	17	18 (5–21)	10/7	49 (3–112)
Wu 2019 [21]	China (SPHIC)	retrospective	Survival, toxicity	2015–2018	21	64 (28–82)	10/11	21.8 (7.2–39.2)

NR, no reported; NIRS, National Institute of Radiological Sciences; GHMC, Gunma University Heavy Ion Medical Center; HIT, Heidelberg Ion Beam Therapy Center; SPHIC, Shanghai Proton and Heavy Ion Center

### Clinical features

The 12 articles mainly included chordomas, chondrosarcomas, and osteosarcomas. The patients in four studies were unresectable [12, 13, 20, 21], eight studies did not receive chemotherapy [12, 13, 15–20], and three studies received radiotherapy alone [12, 13, 20]. The median target volume ranged from 32 to 512.7 cc; the main details of the tumor site, grading, and tumor status (primary, recurrence, and metastasis) are shown in Table 3.

**Table 3:** Clinical features of all included studies

Study	Type of disease	Histology	Grading	Tumor status P/R/M	Tumor site	Median target volume (cc)
Shiba 2021 [10]	Bone Sarcoma	Chordoma = 32; Chondrosarcoma = 9; UPS = 3 Osteosarcoma = 8; SEF = 1	NR	NR	Pelvis = 49; Axis = 4	215.6 (1.6–2074.3)
Mohamad 2018 [11]	Bone Sarcoma	Osteosarcoma = 26	NR	22*/1/3	Pelvic = 24; Axis = 2	452 (172–1774)
Imai et al. 2017 [12]	Bone Sarcoma	Chondrosarcomas = 73	G1 = 14; G2 = 51; G3 = 4 Dedifferentiated = 4	55/17/1	Pelvic = 38; Axis = 35	471 (25–2900)
Imai 2016 [13]	Bone Sarcoma	Chordoma = 188	NR	188/0/0	Sacral	345 (42–1497)
Matsunobu 2012 [14]	Bone Sarcoma	Osteosarcoma = 78	NR	74**/4/0	Spine/Paraspinal = 15 Pelvic = 61; Others = 2	510 (60–2299)
Imai 2011 [15]	Bone Sarcoma	Chordoma = 95	NR	84/11/0	Sacral	370 (47–1468)
Mizoe 2009 [16]	Bone Sarcoma	Chordoma = 33	NR	NR	Skull base	32 (2–328)
Mattke 2018 [17]	Bone Sarcoma	Chondrosarcoma = 79	G1 or G2	70/9/0	Skull base	34.6 (8–133)
Uhl 2014 [18]	Bone Sarcoma	Chordoma = 155	NR	101/54/0	Skull base	70 (2–294)
Uhl 2014 [19]	Bone Sarcoma	Chondrosarcoma = 79	G1 = 51; G1-2 = 7; G2 = 20; G3 = 1	54/25/0	Skull base	60.5 (3–254.4)
Combs 2009 [20]	Bone Sarcoma	Chordoma = 7; Chondrosarcoma = 10	NR	14/3/0	Skull base	73.2 (20.1–182)
Wu 2019 [21]	Bone Sarcoma	Chordoma = 16; Chondrosarcoma = 5	NR	8/13/0	Extracranial = 21	512.7 (142.6–2893)

NR, no reported; P/R/M, primary/recurrent/metastasis; UPS, undifferentiated pleomorphic sarcoma; SEF, sclerosing epithelioid fibrosarcoma

*Two patients were second primary; **Three patients were second primary

### Carbon ion radiotherapy

In terms of the carbon ion radiotherapy, each research center used a different beam-delivery system (Table 4). Passive scanning is mainly performed in Japan, including at the National Institute of Radiological Sciences and Gunma University Heavy Ion Medical Center. Active scanning is mainly performed in China and Europe, including the Shanghai Proton and Heavy Ion Center and the Heidelberg Ion-Beam Therapy Center. Regarding the total dose, each research center used different dose fractionation (Table 4).

**Table 4:** Treatment regimens main results of all included studies

Study	Surgery	Chemotherapy	Beam-Delivery	Total dose (Gy RBE)	Fractions (n)	Dose/fraction Gy (RBE)
Shiba 2021 [10]	7 (13.2%)	6 (11.3%) Osteosarcoma only	NR	64–70.4	16	4.0–4.4
Mohamad 2018 [11]	4 (15%)	26 (100%)	Passive scanning	52.8–73.6	16	3.3–4.6
Imai 2017 [12]	0	0	Passive scanning	64–73.6	16	4.0–4.6
Imai 2016 [13]	0	0	Passive scanning	64–73.6	16	4.0–4.6
Matsunobu 2012 [14]	11 (14.1%)	61 (78.2%)	Passive scanning	52.8–73.6	16	3.3–4.6
Imai 2011 [15]	11 (11.6%)	0	Passive scanning	52.8–73.6	16	3.3–4.6
Mizoe 2009 [16]	33 (100%)	0	Passive scanning	48.0–60.8	16	3.0–3.8
Mattke 2018 [17]	75 (94.9%)	0	Active scanning	60	20	3.0
Uhl 2014 [18]	139 (89.7%)	0	Active scanning	60	20	3.0
Uhl 2014 [19]	67 (84.8%)	0	Active scanning	60	20	3.0
Combs 2009 [20]	0	0	Active scanning	60	20	3.0
Wu 2019 [21]	0	NR	Active scanning	69 (57–80)	18–25	3.2

NR, no reorted; RBE: relative biological effectiveness

### LC rate outcomes of C-ion RT

**Fig. 2:**
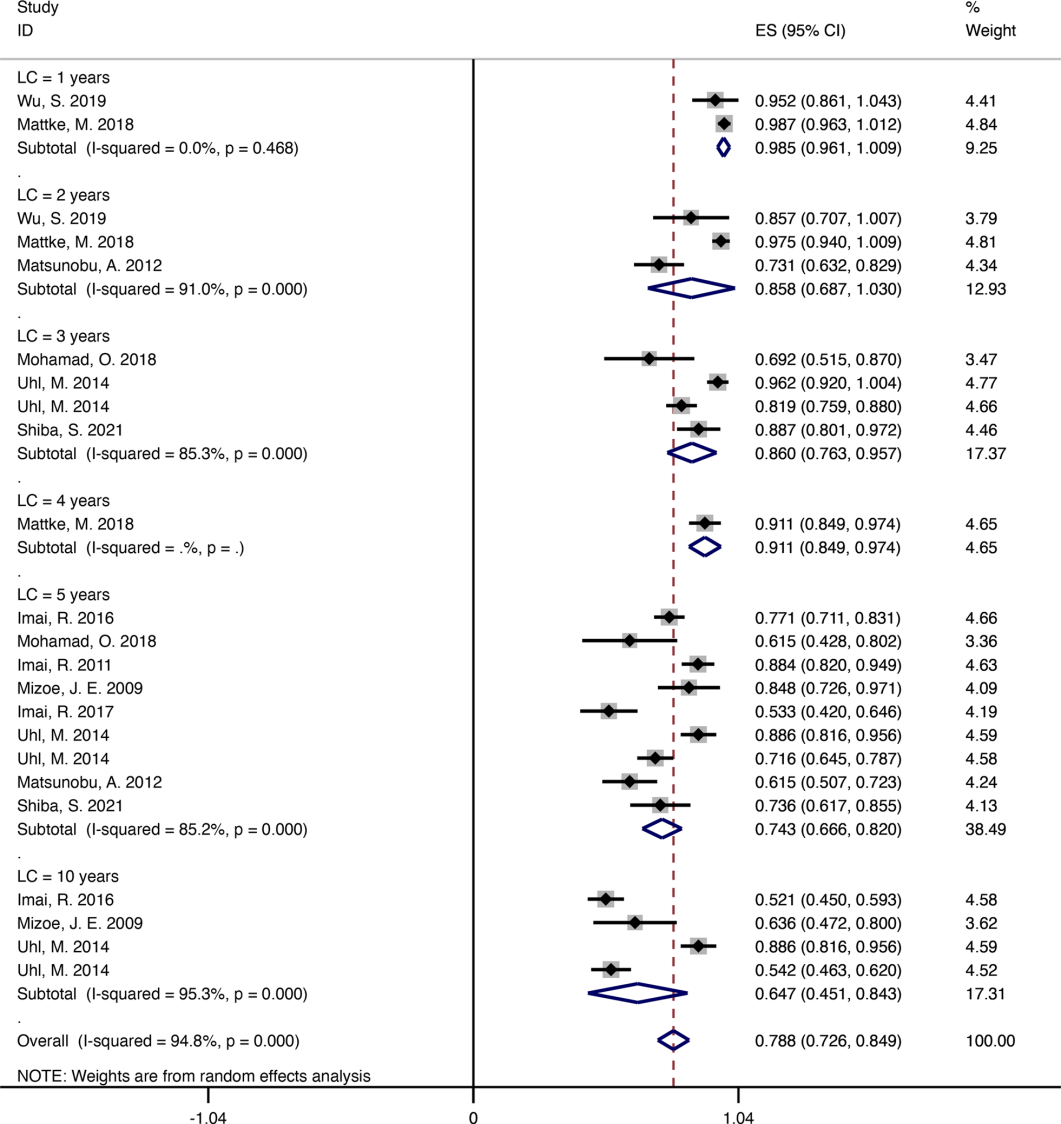
The pooled incidences of local control at 1, 2, 3, 4, 5 and 10 years after C-ion RT for bone sarcomas

These patients mainly had chordomas, chondrosarcomas, and osteosarcoma. In terms of the LC incidence at 1, 2, 3, 4, 5, and 10 years in these studies were 98.5% (95% CI = 0.961–1.009, *I*^2^ = 0%), 85.8% (95% CI = 0.687–1.030, *I*^2^ = 91%), 86% (95% CI = 0.763–0.957, *I*^2^ = 85.3%), 91.1% (95% CI = 0.849–0.974), 74.3% (95% CI = 0.666–0.820, *I*^2^ = 85.2%), and 64.7% (95% CI = 0.451–0.843, *I*^2^ = 95.3%), respectively (Fig. 2) [10–20]. For the four studies regarding chordoma, the LC incidence at 3, 5, and 10 years were 81.9% (95% CI = 0.759–0.880), 80.2% (95% CI = 0.723–0.881, *I*^2^ = 77.6%), and 71.9% (95% CI = 0.621–0.817, *I*^2^ = 0%), respectively (Additional file 1: Fig. S1) [13, 15, 16, 18]. For the three studies regarding chondrosarcoma, the LC incidence at 1, 2, 3, 4, 5, and 10 years were 98.7% (95% CI = 0.963–1.012), 97.5% (95% CI = 0.940–1.009), 96.2% (95% CI = 0.920–1.004), 91.1% (95% CI = 0.849–0.974), 71.3% (95% CI = 0.367–1.058, *I*^2^ = 96.3%), and 88.6% (95% CI = 0.816–0.956), respectively (Additional file 2: Fig. S2) [12, 17, 19]. In addition, for the two studies regarding osteosarcoma, the LC incidence at 2, 3, and 5 years were 73.1% (95% CI = 0.632–0.829), 69.2% (95% CI = 0.515–0.870), and 61.5% (95% CI = 0.522–0.709, *I*^2^ = 0%), respectively (Additional file 3: Fig. S3) [11, 14].

### OS rate outcomes of C-ion RT

**Fig. 3:**
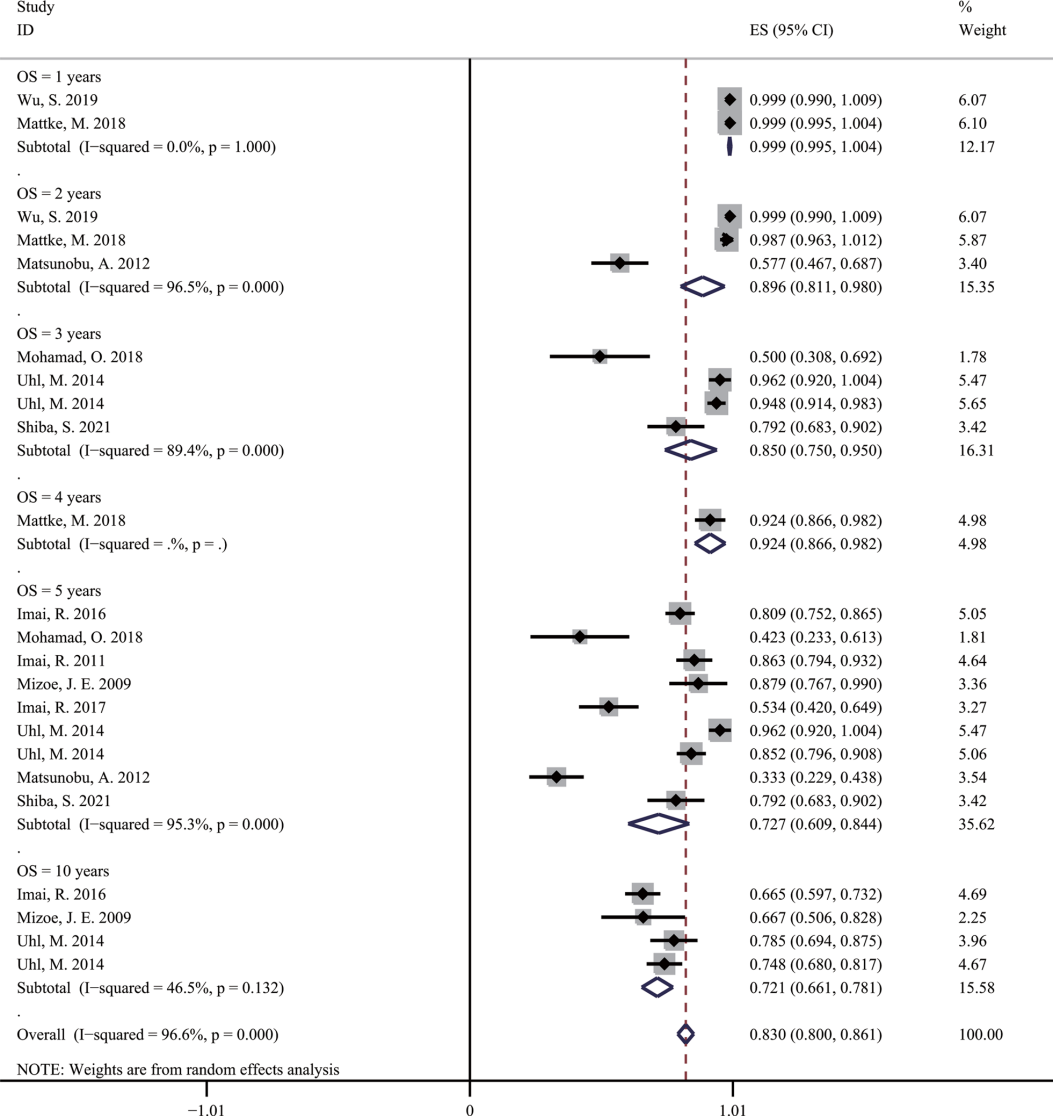
The pooled incidences of overall survival at 1, 2, 3, 4, 5 and 10 years after C-ion RT for bone sarcomas

As shown in Fig. 3, after undergoing C-ion RT for 1, 2, 3, 4, 5, and 10 years, the OS rates for BSs were 99.9% (95% CI = 0.995–1.004, *I*^2^ = 0%), 89.6% (95% CI = 0.811–0.980, *I*^2^ = 96.6%), 85% (95% CI = 0.750–0.950, *I*^2^ = 89.4%), 92.4% (95% CI = 0.866–0.982), 72.7% (95% CI = 0.609–0.844, *I*^2^ = 95.3%), and 72.1% (95% CI = 0.661–0.781, *I*^2^ = 46.5%), respectively [10–21]. For different BSs, the OS rates for chordoma at 3, 5, and 10 years were 94.8% (95% CI = 0.914–0.983), 84.2% (95% CI = 0.809–0.875, *I*^2^ = 0%), and 70.1% (95% CI = 0.640–0.763, *I*^2^ = 35.8%), respectively (Additional file 4: Fig. S4) [13, 15, 16, 18]; the OS rates for chondrosarcoma at 1, 2, 3, 4, 5, and 10 years were 99.9% (95% CI = 0.995–1.004), 98.7% (95% CI = 0.963–1.012), 96.2% (95% CI = 0.920–1.004), 92.4% (95% CI = 0.866–0.982), 75.2% (95% CI = 0.332–1.171, *I*^2^ = 97.9%), and 78.5% (95% CI = 0.694–0.875), respectively (Additional file 5: Fig. S5) [12, 17, 19]; the OS rates for osteosarcoma at 2, 3, and 5 year were 57.7% (95% CI = 0.467–0.687), 50% (95% CI = 0.308–0.692), and 35.4% (95% CI = 0.263–0.446, *I*^2^ = 0%), respectively (Additional file 6: Fig. S6) [11, 14].

### Toxicity

Across all studies, the incidence of acute and late toxicities was mainly grade 1 to grade 2, and grade 1 to grade 3, respectively (Table 5). Acute toxicity grade 3 was observed in two studies, the incidence of which was 3.2–3.8% [14, 15]. Late toxicity grade 4 was observed in five articles, with an incidence of 2.1–8% [11–15]. Sarcomas of the skull base were not observed at more than grade 2 early and late toxicity [16–20]. Two studies of sacral sarcoma discovered grade 4 late toxicity of the skin and sciatic nerve neuropathy; however, the incidence was 1.1–2.1% [13, 15]. Regarding the sarcoma sited in the pelvis, axis, and spine or paraspinal area, three studies observed grade 4 late toxicity of vertebral body compression fractures, fractures, and bone necrosis, the incidence of which was 2.6–6.8% [11, 12, 14].

**Table 5:** Survival outcomes, toxicity incidence and prognostic factors on patients of all included studies

Study	Local recurrence	Metastasis	Local control	Overall survival	Toxicity	Prognostic factors been evaluated
Shiba 2021 [10]	8 (15.1%)	11 (20.8%)	3-year (88.6%) 5-year (73.8%)	3-year (79.7%) 5-year (79.7%)	Acute: ≤ G2 Late: ≤ G3	Chordoma: Age, Sex, Chemotherapy, **Performance status(0–1 or 2–3)**^**c**^, Prior treatment, Distance of tumor-GI(≤ 3 mm or > 3 mm), Distance of tumor-GI(≤ 5 mm or > 5 mm), **GTV volume(≤ 300cm**^**3**^** or > 300cm**^**3**^**)**^**a**^, GTV D98(≤ 64GyE or > 64GyE), GTV D95(≤ 66GyE or > 66GyE), GTV V64(≤ 98 or > 98), GTV V60(≤ 98 or > 98), GTV V < 64(≤ 1cm^3^ or > 1cm^3^), **GTV V < 60(≤ 1cm**^**3**^** or > 1cm**^**3**^**) **^**a**^
Mohamad 2018 [11]	2 (7.7%)	14 (53.8%)	3-year (69.9%) 5-year (62.9%)	3-year (50%) 5-year (41.7%)	Acute: No Late: ≤ G4(G4 = 8%)	Age, Sex, Performance status(1 or 2), Pathologic subtype(Osteoblastic or Others), Tumor location(Pelvis or Others), Tumor status(Primary or Others), Tumor status(Non-metastatic or Metastatic), Target volume(< 452cm^3^ or ≥ 452cm^3^), **Largest tumor diameter**(**≤ 9.5 cm or > 9.5 cm**)^**ab**^, Response to chemotherapy(SD + PR or Others*), Dose(≥ 70.4GyE or < 70.4GyE)
Imai 2017 [12]	Unclear	NR	5-year (53%)	5-year (53%)	Acute: NR Late: ≤ G4(G3 = 4%, G4 = 7%)	Different grade groups(G1 or G2), **Tumor size(cut-off of 470 cm**^**3**^**) **^**abe**^, **Different grade groups(G1 or G3 anddedifferentiated) **^**be**^, **Different grade groups(G2 and dedifferentiated) **^**be**^, Different grade groups(G1 or G2), Tumor status(primary or recurrenc), Tumor location (spine and other, pelvis), Tumor status (primary, recurrence and metastasis), Age (cut-off of 65 years)
Imai 2016 [13]	41 (21.8%)	54 (28.7%)	5-year (77.2%) 10-year (52%)	5-year (81.1%) 10-year (66.8%)	Acute: NR Late: ≤ G4(G4 = 1.1%)	Sex, Tumor volume(≤ 500cm^3^ or > 500cm^3^), Level of proximal invasion(≥ S2 or < S2), Total irradiated dose(≤ 67.2GyE or > 67.2GyE)
Matsunobu 2012 [14]	21 (26.9%)	41 (52.6%)	2-year (73%) 5-year (62%)	2-year (58%) 5-year (33%)	Acute: ≤ G3(G3 = 3.8%) Late: ≤ G4(G3 = 5.1%, G4 = 3.8%)	Age, Sex, **Performance status(1 or 2)**^**ab**^, Tumor site(Pelvis or Others), Pathologic subtype, Tumor status(Primary tumor or Metastatic tumor), **Clinical target volume(< 500cm**^**3**^** or ≥ 500cm**^**3**^**)**^**ab**^, **ALP(Normal or ≥ 335 IU/L)**^**b**^, **CRP(Normal or ≥ 0.3 mg/dL)**^**b**^, Prior surgery, Prior chemotherapy, Total dose(> 70GyE or ≤ 70GyE)
Imai 2011 [15]	6 (6.3%)	NR	5-year (88%)	5-year (86%)	Acute: ≤ G3(G3 = 3.2%) Late: ≤ G4(G3 = 2.1%, G4 = 2.1%)	NR
Mizoe 2009 [16]	Unclear	NR	5-year (85.1%) 10-year (63.8%)	5-year (87.7%) 10-year (67%)	Acute: ≤ G2 Late: ≤ G2(G2 = 3%)	Age, sex, KPS, dose, Gross tumor volume (GTV)
Mattke 2018 [17]	5 (6.3%)	0	1-year (98.6%) 2-year (97.2%) 4-year (90.5%)	1-year (100%) 2-year (98.5%) 4-year (92.9%)	Acute: ≤ G2 Late: ≤ G2	Age, Sex, Tumor volume(≤ 36.6 cm^3^ or > 36.6 cm^3^), Tumor status (Primary/recurrent)
Uhl 2014 [18]	55 (35.5%)	4 (2.6%)	3-year (82%) 5-year (72%) 10-year (54%)	3-year (95%) 5-year (85%) 10-year (75%)	Acute:NR Late: Quantitative toxicity results	**PTV volume(< 100 ml or ≥ 100 ml) **^**a**^**, Total dose(≤ 51 GyE or > 51 GyE) **^**a**^
Uhl 2014 [19]	10 (12.7%)	NR	3-year (95.9%) 5-year (88%) 10-year (88%)	3-year (96.1%) 5-year (96.1%) 10-year (78.9%)	Acute:NR Late: Quantitative toxicity results	**Age(< 45 years) **^**a**^**, Boost volume(≤ 55 ml) **^**a**^, Sex, Dose, Tumor grade(grade 1 or grade 2), Time of treatment(primary or recurrenc)
Combs2009 [20]	1 (5.9%)	0	Crude local control rate was 94%	Crude overall survival rate was 100%	Acute: ≤ G2(G2 = 6%) Late: No	NR
Wu 2019 [21]	3 (14.3%)	4 (19.0%)	1-year (93.8%) 2-year (85.2%)	1-year (100%) 2-year (100%)	Acute: ≤ G1(G1 = 48%) Late: ≤ G1	Age, Metal implantation, Sex, Treatment (primary or recurrence), Dose, Tumor volume

Bold was defined as a statistically significant prognostic factor (*p* ≤ 0.05)

NR, no reported

^a^Factor significantly correlated with local control (LC) (*p* ≤ 0.05); ^b^factor significantly correlated with overall survival (OS) (*p* ≤ 0.05); ^c^factor significantly correlated with progress-free survival (PFS) (*p* ≤ 0.05); ^d^factor significantly correlated with distance Progress-free survival (DPFS) (*p* ≤ 0.05);

^e^Factor significantly correlated with disease free survival (DFS) (*p* ≤ 0.05); ^f^factor significantly correlated with local recurrence (LR) (*p* ≤ 0.05); *Others include progressive disease and incomplete chemotherapy regimen; excluding unknown response

### Prognostic factors of C-ion RT effectiveness

In our systematic review, 10 studies reported the prognostic factors of *C-ion RT* effectiveness. The following factors were evaluated: age, sex, performance status, pathology, histological grading, tumor status, tumor location, target volume, chemotherapy, and total dose. Table 5 shows the main details of the prognostic factors of *C-ion RT* effectiveness in all the included studies.

## Discussion

In our systematic review, BSs patients, including patients with chordomas, chondrosarcomas, and osteosarcomas, were treated with C-ion RT. The prescribed doses were 48 to 80 Gy RBE for BSs (Table 4). The 3-year and 5-year OS rates were 85% and 72.7%, respectively (Fig. 3), the 3-year and 5-year LC rates were 86% and 74.3% (Fig. 2), respectively [10–21]. According to previous clinical outcomes in X-ray RT, the 5-year OS and LC rates were 50–70% and 27–67%, respectively, and those in proton RT were 67–84% and 62–81%, respectively [5, 6, 22–29]. Therefore, compared with those in past clinical reports, the efficacy and safety of C-ion RT for bone sarcoma were comparable to those of proton RT but better than those of X-ray RT. Moreover, patients treated with C-ion RT had similar surgical outcomes despite being unsuitable for surgery [5, 30–35]. In this case, C-ion RT may be an important local treatment option for patients with such BSs.

In the four studies regarding chordoma with C-ion RT in our study (Additional file 1: Fig. S1 and Additional file 4: Fig. S4), the LC rates at 3, 5, and 10 years were 81.9%, 80.2%, and 54.1%, respectively; the OS rates at 3, 5, and 10 years were 94.8%, 84.2%, and 70.1%, respectively [13, 15, 16, 18], Due to the low possibility of metastasis, complete surgical resection or control of local tumor progression is a critical factor for long-term survival [36–38]. Both base skull and sacrococcygeal chordomas are often adjacent to important neuroaxes; therefore, complete resection is often difficult to achieve. According to the previous reports, the proportion of complete resection of the tumor was approximately 20–70%, LC rate of total resection was approximately 60–80%, and LC rate of subtotal resection was approximately 25–50% [39–42]. In terms of proton therapy alone, Chen et al. reported a study including 24 unresectable chordomas, with 19 sacral chordomas [43]. They irradiated the tumor with a median total dose of 77.4 Gy (RBE). The 5-year local progression-free survival and OS rates were 79.8% and 78.1%, respectively. In a systematic review by Amichetti et al. [44], the mean 5-year LC and OS rates after proton therapy were 69% and 80%, respectively. Overall, carbon ion therapy for chordoma had outcomes similar to those of surgical and proton radiotherapy. In addition, an adequate total dose is essential for the LC of chordomas with carbon ion therapy. Uhl et al. prescribed a total dose of 60.0 Gy (RBE), with LR and 5-year LC rates of 35.5% and 72%, respectively [18]. The clinical results were inferior to those of three studies from Japan [13, 15, 18]. Nevertheless, the LR rate was 6.3–35.5% with carbon ion therapy for chordoma in our four selected studies [13, 15, 18], which was still significantly lower than the LR rate of 35–50% after primary chordoma surgery [36–38].

Surgical treatment is the first choice of treatment for chondrosarcoma. However, chondrosarcomas located in the base skull or spine/paraspinal region are often difficult to completely resect, and even if resection can be performed, there is still a risk of recurrence. According to Bloch et al., the 5-year LR rate was 44% after surgery alone, 19% after radiotherapy alone, and 9% after surgery combined with adjuvant RT [45, 46]. In this case, RT may be an important therapeutic strategy for chondrosarcomas that are unresectable or residual after incomplete surgery. Owing to the radiation resistance of chondrosarcomas, a relatively high dose is required to achieve an adequate LC rate. Kano et al. used a Gamma knife to irradiate base skull chondrosarcomas. According to this report, the median target volume and margin dose were 8 cm^3^ and 15 Gy, respectively, and the LC rates at 3, 5, and 10 years were 88%, 85%, and 70%, respectively [47]. In terms of proton therapy, Munzenrider et al. prescribed a median dose of 72 Gy (RBE) to irradiate G1 chondrosarcomas, in a study including 225 patients, and reported that the LC rate at 5 and 10 years was 98% and 94%, respectively [48]. Feuvret reported that in 159 patients who received proton therapy alone or with a combination of protons and photons, with a median dose of 70.2 Gy (RBE), the LC rate at 5 and 10 years was 96.4% and 93.5%, respectively [49]. Hug et al. published a study of 25 patients after proton radiotherapy, wherein the 5-year LC rate was 92% [50]. Weber et al. reported a 7-year LC rate of 93.6% for patients with chondrosarcomas treated with proton therapy after surgery [51]. Our systematic evaluation included three studies regarding chondrosarcomas managed with carbon ion therapy. Mattke et al. published clinical results of carbon ion therapy alone for 79 patients with skull base chondrosarcomas, the LC rate at 1, 2, and 4 years was 98.6%, 97.2%, and 90.5%, respectively [17]. A study by Uhl et al. showed 79 patients after carbon ion therapy alone with a dose of 60 Gy (RBE); the LC rate at 3-, 5-, and 10-years were 95.9%, 88%, and 78.9%, respectively [19]. However, a study reporting a 5-year LC rate of 53% for 73 patients after C-ion RT alone with a dose of 64–73.6 Gy (RBE) was published by Imai et al. [12]. The efficacy of C-ion RT was worse than that of surgery. The most likely reason for this was that chondrosarcomas were present close to the spinal cord or sacral lesions. Another suggested reason was that these patients were older than those in the groups undergoing surgery [52–54].

According to the Cooperative Osteosarcoma Study report, which included 67 patients with pelvic osteosarcoma, the LC and OS rates at 5 years were 30% and 27%, respectively. However, the LC and OS rates at 5 years were 6% and 0%, respectively, which are unsuitable for surgery patients [30]. Osteosarcoma is relatively radiation-resistant to conventional radiotherapy. Especially in patients with pelvic and axial osteosarcoma, it is difficult to administer high-dose radiation to the tumor because it is adjacent to the intestinal tract and spinal cord. However, particle radiotherapy, especially carbon ion therapy, has unique physical and biological advantages [55, 56]. We included two studies regarding osteosarcoma that utilized carbon ion therapy: the LC incidence at 2, 3, and 5 years was 73.1%, 69.2%, and 61.5%, respectively (Additional file 3: Fig. S3); the OS rates at 2, 3, and 5 years were 57.7%, 50%, and 35.4%, respectively (Additional file 6: Fig. S6); and the LR incidence was 7.7% to 26.9% (Table 5) [11–14]. Regarding proton therapy, a study by DeLaney et al. reported unresectable or incompletely resected truncal osteosarcomas. Patients who received proton radiotherapy have a lower risk of recurrence after incomplete resection [57]. In another report, a proton or mixed proton/photon radiotherapy was performed for osteosarcomas of the trunk; the LC and OS rates at 5-years were 72% and 67%, respectively [27]. This survival rate appears to be superior to that of carbon ion radiotherapy. The most likely reason for this was that the baseline characteristics (stage, resectability, site, grade, and size) in this study were more favorable. In terms of the LC rate, carbon ion therapy for pelvic or truncal osteosarcoma showed similar proton outcomes but was superior in terms of surgical outcomes despite including patients who had more unfavorable baseline characteristics. It is well known that distant metastasis is the major factor affecting the OS rate of osteosarcoma. Because of the great differences in systemic treatment in different studies, the reported OS rates are significantly different.

Regarding toxicity, the incidence of acute and late toxicity was mainly grade 1 to grade 2 and grade 1 to grade 3, respectively. Regarding the acute toxicity, grade 3 was observed in two studies, with an incidence of 3.2–3.8% [14, 15]. The most common event was an acute skin reaction [10, 11, 14–17, 20], and a grade 3 skin acute reaction was observed in six patients [14, 15]. No grade 4 or higher skin and mucosal acute reactions were observed in any of the studies. Kamada et al. considered that the maximum tolerated dose for patients with no subcutaneous tumor and subcutaneous tumor involvement may be 73.6 Gy (RBE) and 70.4 Gy (RBE) or less [58]. In terms of the late toxicity, grade 4 was observed in five articles, with an incidence of 1.1% to 8% [11–15]. The BSs of the skull base not observed at more than grade 2 early and late toxicities [16–20]. The two studies of sacral sarcoma discovered grade 4 late toxicity of the skin and sciatic nerve neuropathy; however, the incidence was 1.1–2.1% [13, 15]. In a study by Yanagi et al., the area of skin irradiated with > 60 Gy (RBE) (S60 > 20 cm^2^) was the most important factor for grade 4 skin late toxicity development [59]. Imai et al. indicated that the risk factors for sciatic nerve injury in sacral chordoma may be the length (> 10 cm) and dose (> 70 Gy (RBE)) of irradiation [15]. Regarding sarcoma located in the pelvis, axis, and spinal or paraspinal area, three studies observed grade 4 late toxicity of vertebral body compression fractures, fracture, and bone necrosis, with an incidence of 2.6–6.8% [11, 12, 14]. Although the toxicity of carbon ion therapy was low and acceptable, late toxicity required larger samples and long-term follow-up.

In our systematic review, there were 10 studies that reported the prognostic factors of C-ion RT effectiveness (Table 5) [10–14, 16–19, 21]. The following factors were evaluated: age, sex, performance status, pathology, histological grading, tumor status, tumor location, target volume, chemotherapy, and total dose. Prognostic factors varied widely among the selected studies. Overall, most studies have shown that the target volume is a common significant prognostic factor for BSs. Furthermore, younger age, better performance status, and a higher total dose were significantly associated with better LC and OS.

This systematic review and meta-analysis had several limitations. First, gray literature was not included, and there may be publication bias. Second, the results of our search showed that 58% of the literature on C-ion RT for BSs was from Japan, 33% of the literature was from Germany, and one study was from China. Therefore, there could be a reporting bias. In addition, all the studies were case series reports without randomized controlled studies and included small sample sizes, which would affect the reliability of the conclusions of this systematic review. However, all study designs were reasonable, the missed follow-up rates were low, and the strength of the endpoints was high, with all studies evaluating the OS and LC as specific outcomes.

As an advanced radiotherapy technique, carbon ion therapy has shown promising efficacy and acceptable toxicity in BSs. However, there are still some areas of insufficient carbon ion radiotherapy for BSs. First, previous studies on carbon ion therapy have often involved various types of BSs. Different pathological types of BSs may have inconsistent optimal dose patterns, and individualized carbon ion radiotherapy still requires further study. Second, although carbon ion therapy for BSs has achieved a good LC rate, integrated treatment modalities, including chemotherapy, antiangiogenic therapy, and immunotherapy, require further study. Third, the number of patients treated with carbon ions for BSs was too small, although a potential role of carbon ions in improving LC at low toxicity was found. Finally, whether carbon ion radiotherapy is superior to other radiotherapy technologies needs to be determined in high-quality prospective, randomized controlled clinical trials in bone sarcoma patients.

## Conclusion

As one of the more advanced radiotherapy technology, C-ion RT is promising for patients who have BSs that is unresectable or residual after incomplete surgery. The data indicated that C-ion RT was safe and effective for BSs, showing promising results in local control, overall survival, and acceptable acute and late toxicity. However, whether carbon ion radiotherapy is superior to other radiotherapy technologies needs to be performed in high-quality prospective, randomized controlled clinical trials.

## Supplementary Information

**Additional file 1: Fig. S1.** The pooled incidences of local control at 3, 5 and 10 years after C-ion RT for chordoma.

**Additional file 2: Fig. S2.** The pooled incidences of local control at 1, 2, 3, 4, 5 and 10 years after C-ion RT for chondrosarcoma**.**

**Additional file 3: Fig. S3.** The pooled incidences of local control at 2, 3 and 5 years after C-ion RT for osteosarcoma.

**Additional file 4:Fig. S4.** The pooled incidences of overall survival at 3, 5 and 10 years after C-ion RT for chordoma.

**Additional file 5: Fig. S5.** The pooled incidences of overall survival at 1, 2, 3, 4, 5 and 10 years after C-ion RT for chondrosarcoma.

**Additional file 6: Fig. S6.** The pooled incidences of overall survival at 2, 3 and 5 years after C-ion RT for osteosarcoma.

## Data Availability

All data are provided.

## Abbreviations

C-ion RT: Carbon ion radiotherapy
BSs: Bone sarcomas
LR: Local recurrence
RT: Radiotherapy
LC: Local control
OS: Overall survival
RBE: Relative biological effectiveness
PRISMA: Preferred Reporting Items for Systematic Reviews and Meta-analysis
JBI: Joanna Briggs Institute
RE: Random effects
Cis: Confidence intervals
